# Male infertility: what on earth is going on? Pilot international questionnaire study regarding clinical evaluation and fertility treatment for men

**DOI:** 10.1530/RAF-22-0033

**Published:** 2022-09-26

**Authors:** Nkoyenum Pamela Olisa, Lisa Campo-Engelstein, Sarah Martins da Silva

**Affiliations:** 1 Reproductive Medicine Research Group, School of Medicine, Ninewells Hospital and Medical School, University of Dundee, Dundee, UK; 2 Institute for the Medical Humanities, Preventative Medicine and Population Health, University of Texas Medical Branch, Galveston, Texas, USA

**Keywords:** male infertility, sperm, assisted reproduction technology, ART, intracytoplasmic sperm injection, ICSI

## Abstract

Infertility is a time-consuming and exhaustive process, which disproportionally affects women. Although concerns have been raised about deficiencies in the clinical evaluation of infertile men, there are currently little published data documenting this. A SurveyMonkey questionnaire was therefore created to capture the current clinical practice of fertility specialists working in *in vitro* fertilisation clinics. Responses were collected from May to July 2021. A total of 112 clinicians completed the pilot survey with respondents from Europe (*n* = 49; 43.8%), Africa (*n* = 39, 34.8%), North America (*n* = 6; 5.4%), Asia (*n* = 16; 14.3%), South America (*n* = 1; 0.9%) and Australasia (*n* = 1; 0.9%). Forty-one percent of fertility specialists (45/110) reported taking only a brief medical history and 24% reported that they never routinely examined infertile male patients. Fifty-four percent of fertility specialists also reported issues getting men to undertake diagnostic semen analysis. Treatment for male infertility spanned assisted reproductive technology (ART), with themes of individualised medicine influencing treatment recommendations. Of the clinicians, 48.2% clinicians reported using empirical medical therapy for unexplained male infertility. Notably, 3.6% respondents recommended testosterone treatment, despite the likely negative impact on spermatogenesis. However, high levels of opportunistic general health advice were reported, including discussion of life exposures thought to be important for male reproductive health. This study adds novel evidence and highlights current deficiencies in clinical practice relating to male infertility. Evaluation of the infertile male using simple medical tools (detailed history taking and clinical examination) has the potential to identify treatable or reversible conditions and should be an immediate focus for education and improvement in reproductive medicine. Investment in research and development is much needed in the field of andrology to develop effective non-ART treatment options for male infertility.

**Lay summary:**

Poor sperm quality (male infertility) significantly reduces the chance of natural conception and accounts for half of all cases of infertility, yet affected men are frequently overlooked when couples seek fertility investigations and treatment. Despite a growing awareness of men’s issues and a need to improve patient experience, there is very little documented about how fertility specialists (clinicians) routinely assess and treat male infertility. This study used a SurveyMonkey® questionnaire to capture current clinical practice, with 112 respondents from around the world. Forty-one percent of clinicians did not routinely consider male medical history in detail and 24% never routinely examined infertile men. This should be a focus for improvement in clinical care. As expected, fertility treatment recommended for male infertility was mostly* in vitro* fertilisation and intracytoplasmic sperm injection, where a single sperm is injected into each mature egg. However, 48.2% of clinicians also reported prescribing unproven medical therapy for unexplained male infertility. Of concern, a few clinicians routinely recommended testosterone treatment, which is likely to harm sperm production. However, advice regarding general health was universally delivered.

## Introduction

Infertility is a global health problem. Its effects are largely unseen, yet fertility problems have a profound impact on psychological well-being and quality of life (Nussbaum 2011, Luk & Loke 2015). Wider repercussions often further compound the misery of infertility, including relationship breakdown and divorce, as well as economic deprivation, social stigma and lack of community status in certain cultures (Greil *et al.* 2010). There are no truly reliable figures for the prevalence of infertility. However, best estimates indicate 8–12% of couples of reproductive age are affected (Datta *et al.* 2016, Vander Borght & Wyns 2018) with upwards of 48 million couples experiencing infertility globally (Mascarenhas *et al.* 2012). Higher rates of infertility paradoxically tend to exist in African and South Asian countries where fertility rates are high; a phenomenon termed ‘barrenness amid plenty’ (Inhorn & Patrizio 2015). Recent epidemiological studies report the prevalence of infertility as high as 25% in China (Zhou *et al.* 2018) and 31.1% in Nigeria (Polis *et al.* 2017).

Fertility care encompasses the prevention, diagnosis and treatment of infertility and is an important part of addressing the right of individuals and couples to create a family (Zegers-Hochschild *et al.* 2013). Yet equal and equitable access to fertility care remains a challenge, especially in low- and middle-income countries where personnel, equipment and infrastructure may be limited, or treatment costs prohibitive. Placing both partners at the centre of fertility care is clearly paramount. Nonetheless, evaluation of female fertility has traditionally been the driver for infertility workup, with men frequently overlooked. Importantly, answers to fundamental questions regarding prevention, management and consequences of male infertility are currently unknown, and there is therefore a common perception that reproductive medicine has little to directly offer men. Yet the desire to be a parent is just as strong for men as for women, with research indicating lower long-term mental health for men who do not have children compared to those who become fathers (Fisher *et al.* 2010). Furthermore, the limited available evidence regarding men’s experiences with involuntary childlessness indicates long-term grief and lower quality of life, as well as feelings of loss, depression, exclusion, isolation and risk-taking behaviour (Fisher & Hammarberg 2012, Wischmann & Thorn 2013). A survey of males in the United States found that they rated the importance of being a parent as high (8.5/10). Notably, men were more likely than women to think it preferable to have children than to stay childless and less likely to believe that life could be meaningful without children (Vayena *et al.* 2002).

While data show the need for greater engagement of men in their reproductive health from early adult life onwards (Barratt *et al.* 2021), there is also an argument that interaction with fertility specialists may be an opportunity lost. Male factor accounts for at least half of all cases (Agarwal *et al.* 2015, Kumar & Singh 2015), yet men from infertile couples often do not undergo comprehensive clinical evaluation (Eisenberg *et al.* 2013). Men are less likely to know about variables that affect fertility, including female age, obesity and smoking (Hammarberg *et al.* 2013) and are more likely to overestimate the chance of natural conception or success of fertility interventions (Hammarberg *et al.* 2017). Unfortunately, robust and effective therapeutic interventions to directly treat male infertility are currently lacking, which both limits treatment options and, arguably, delivery of astute clinical care (Duffy *et al.* 2020). The only option for couples is assisted reproduction technology (ART), which is expensive, invasive and without guarantee of success (25–30% live birth rate (LBR) per treatment) (Martins da Silva *et al.* 2017). In the absence of alternative valid approaches, the use of empirical medical therapy (EMT) for men is perhaps not unreasonable, yet at best, this may have limited efficacy and at worst, may do more harm than good (Thaker *et al.* 2020).

The current reality is that there is a lack of clinical expertise (perceived or otherwise) focused on male infertility (Das *et al.* 2020) and a growing voice highlighting a crisis in male reproductive health, including the Male Reproductive Health Initiative (De Jonge & Barratt 2019, Barratt *et al.* 2021). However, there is surprisingly limited survey/questionnaire data published regarding current clinical practice, evaluation and management of men attending specialist fertility centres. A nationwide survey of urology specialists in Japan featured 39 respondents and focused on the aetiology of male infertility and surgical intervention (Yumura *et al.* 2018), the American Urological Association survey of 164 urologists included only 29 reproductive urologists and focused on EMT practices for idiopathic male infertility (Thaker *et al.* 2020) and a recent global survey of reproductive specialists investigated utilisation of oxidative stress testing and use of antioxidants for male infertility (Agarwal *et al.* 2021). In a bid to address this, we conducted a SurveyMonkey questionnaire pilot study to capture a snapshot of delivery of clinical care and current professional opinion from fertility clinicians working in a variety of global ART settings.

## Materials and methods

A SurveyMonkey questionnaire was created (Supplementary Table 1, see section on supplementary materials given at the end of this article). This comprised ten questions asking the location (country) of the clinician, the number of ART cycles performed by their clinic each year and details of current clinical practice, including how men were routinely evaluated, diagnostic semen analysis, treatment approaches and recommendations, lifestyle advice and counselling support. The questionnaire was primarily shared via social media platforms (Facebook, Twitter for professionals, LinkedIn). No mailing lists were used; however, the SurveyMonkey link was also shared with health professionals by personal email contact from the authors, as well as WhatsApp and Telegram obstetrics and gynaecology groups in Nigeria. Responses were retrieved from SurveyMonkey and data were analysed using Excel.

## Results

**Figure 1:**
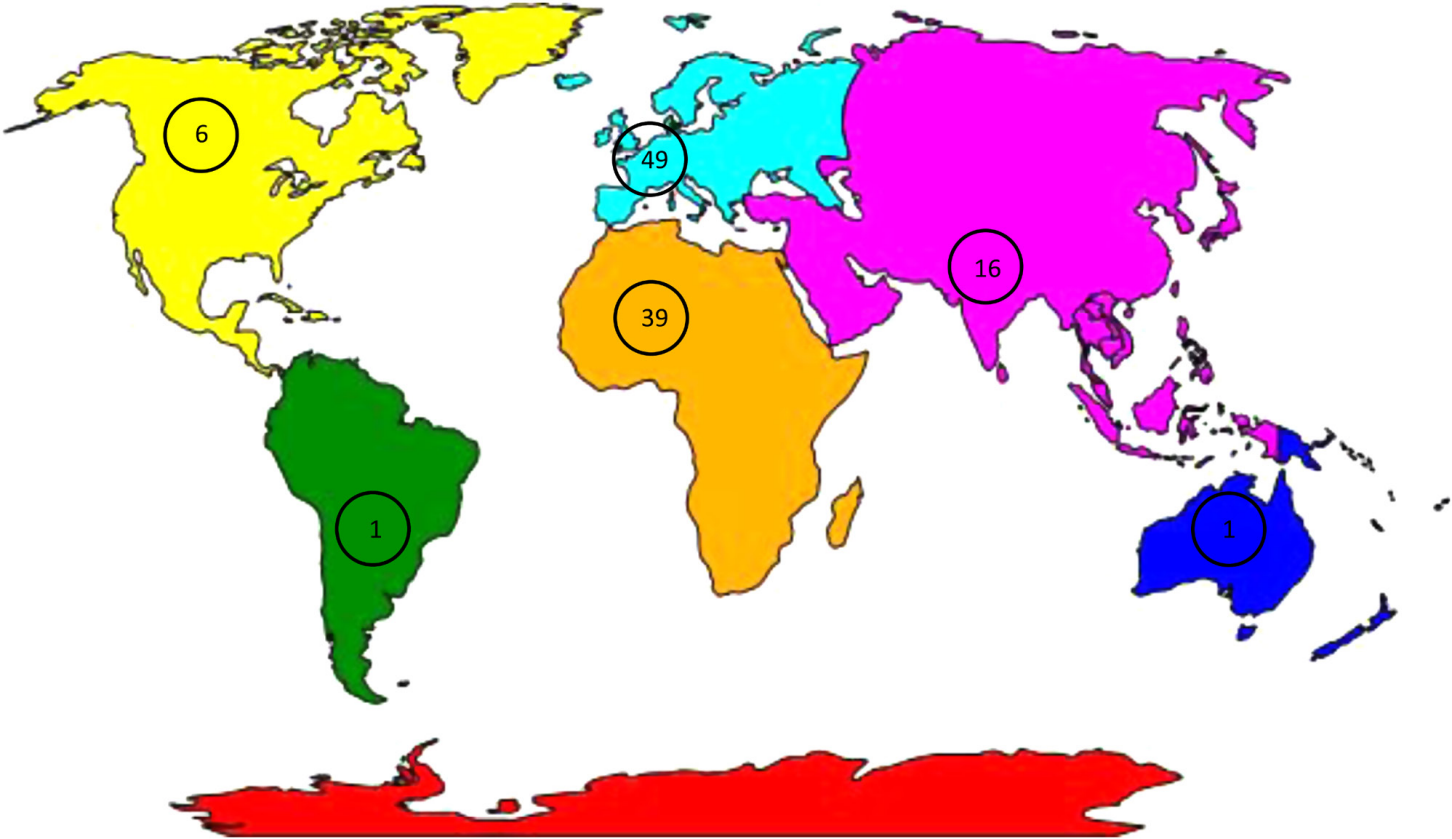
World map depicting distribution of respondents. Number of responses from each continent indicated (Europe (blue) *n*  = 49, Africa (orange) *n*  = 39, North America (yellow) *n*  = 6, South America (green) *n*  = 1, Australasia (dark blue) *n*  = 1 and Asia (pink) *n*  = 16).

In total, 112 clinicians completed the survey, with at least 1 representative from each continent (excluding Antarctica). Europe accounted for 43.8% (*n* = 49) of the respondents, predominantly UK, 34.8% (*n* = 39) of the respondents were from Africa, 5.4% (*n* = 6) were from North America, 0.9% (*n* = 1) from South America, 14.3% (*n* = 16) were from Asia and 0.9% (*n* = 1) from Australasia (Fig. 1). Of the participants, 34.8% reported working in a clinic performing less than 100 ART treatment cycles per year, while 42.9% performed above 500 ART treatment cycles per year. Of the respondents, 9.8% and 12.5% performed between 100–250 and 250–500 ART treatment cycles per year, respectively (Supplementary Fig. 1).

**Figure 2:**
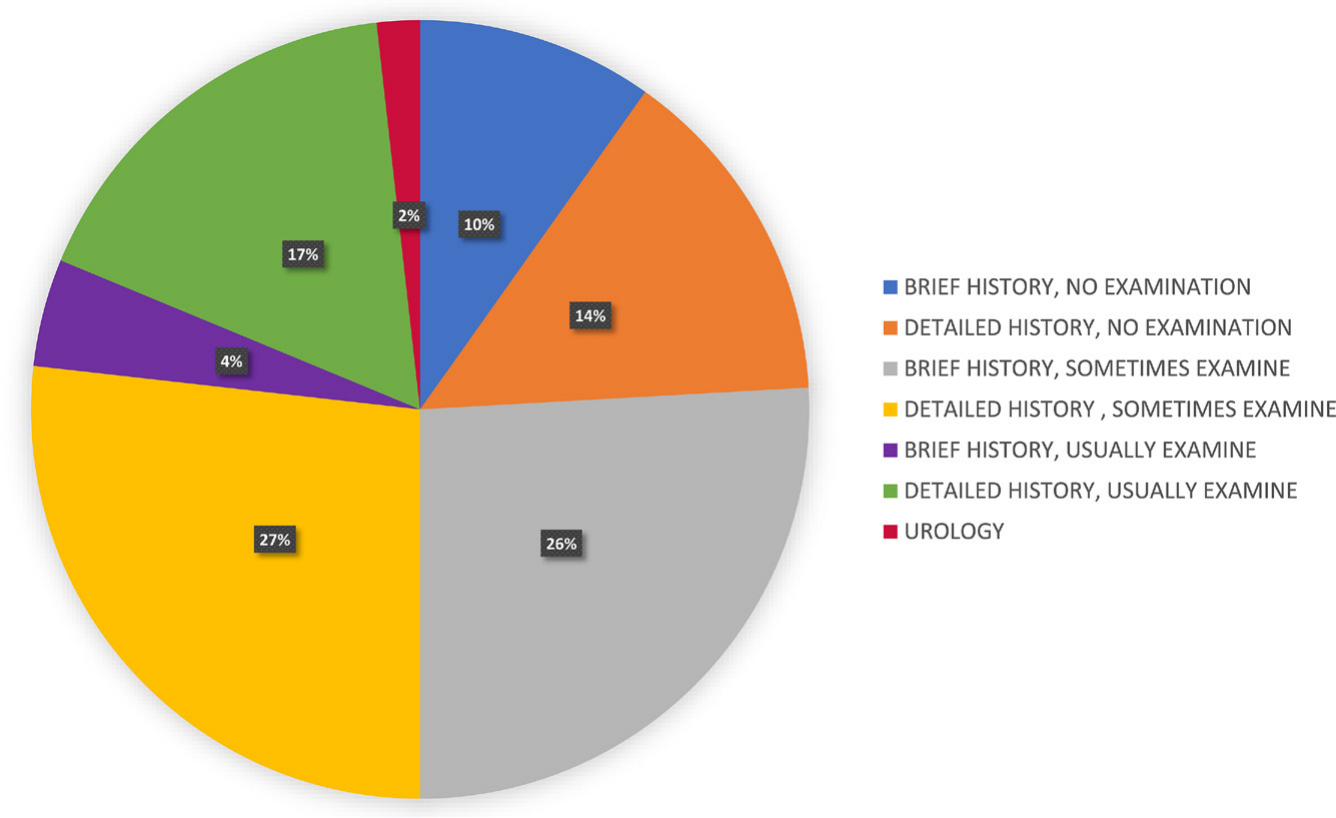
Reported routine evaluation of men in specialist fertility clinic. Brief medical history and no examination (blue) *n*  =  11; detailed medical history and no examination (orange) *n*  = 16; brief medical history and sometimes examination (grey) *n*  = 29; detailed medical history and sometimes examination (yellow) *n*  = 30; brief medical history and usually examination (purple) *n*  = 5; detailed medical history and usually examination (green) *n*  = 19 and evaluation by urology (red) *n*  = 2.

When asked how men are routinely evaluated in the clinic, two respondents (1.8%) indicated that male patients were usually seen by urology specialists. As expected, most men were therefore solely evaluated by fertility specialists. However, only 21.4% (*n* = 24) of fertility specialists reported routine physical examination of male patients coupled with either a brief (*n* = 5) or detailed medical history (*n* = 19). Most ART clinicians (52.7%; *n*  = 59) reported examination of men only sometimes following either a brief (*n* = 29) or detailed (*n* = 30) clinical history. Alarmingly, 24.1% (*n* = 27) clinicians reported that they never examined male patients, with 40.7% (*n* = 11) taking a brief medical history only (Fig. 2).

**Figure 3:**
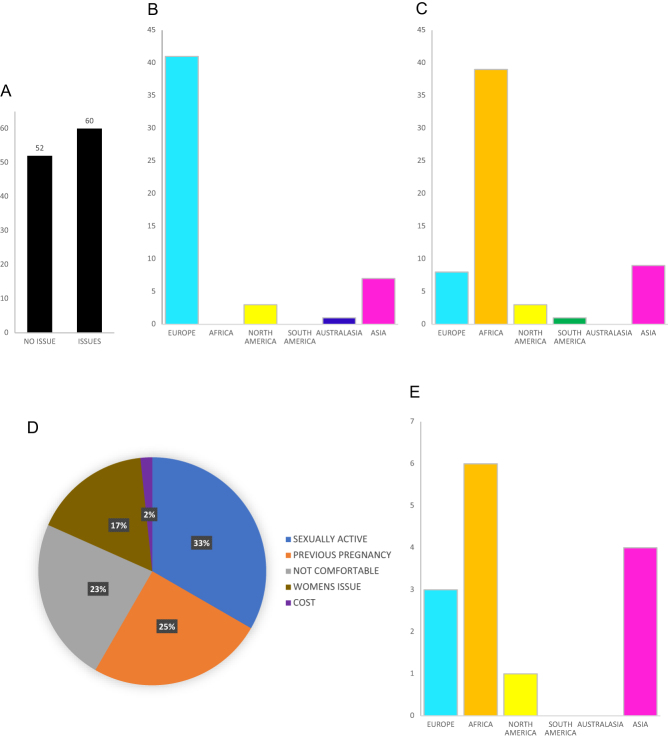
Clinicians’ experiences of getting men to undertake diagnostic semen analysis. (A) No issues were experienced by 46% (*n* = 52) respondents; however, 54% (*n* = 60) reported issues commonly encountered. (B) Geographical distribution of respondents reporting no issues encountered with getting men to undertake diagnostic semen analysis. (C) Geographical distribution of respondents commonly encountering issues getting men to undertake diagnostic semen analysis. (D) Primary reasons reported for issues getting men to undertake diagnostic semen analysis included an assumption by men that they had no fertility problem because they were sexually active (blue; *n*  = 20) or because they had previously fathered a pregnancy (orange; *n*  = 15), that men were not comfortable producing or submitting a sample for testing (grey; *n*  = 14), an assumption that infertility is a woman’s issue (brown; *n*  = 10) as well as cost (purple; *n*  = 1). (E) Geographical distribution of respondents commonly experiencing issues getting men to undertake diagnostic semen analysis because they were uncomfortable producing or submitting a sample for laboratory assessment.

Regarding male fertility testing, 46% of clinicians reported no issues getting men to undertake diagnostic semen analysis (Fig. 3A). Most of these respondents (79%) worked in Europe (Fig. 3B). In contrast, 54% of clinicians commonly encountered issues getting men to undertake semen analysis. Many of these respondents (65%) worked in Africa (Fig. 3C). Reasons cited were varied (Fig. 3D) but included an assumption by men that they had no fertility problem because they were sexually active (33%) or because they had previously fathered a pregnancy (24%), an assumption by men that infertility is a woman’s issue (17%), as well as cost (2%). However, 24% of ART clinicians that reported issues arranging fertility assessment identified that men were uncomfortable producing or submitting a sample for diagnostic semen analysis. Notably, the geographical distribution of these responses was mixed (Fig. 3E), indicating a shared experience by men from heterogeneous backgrounds.

**Figure 4:**
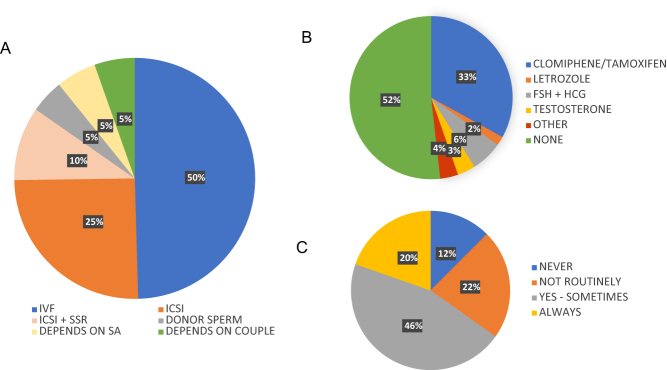
Treatment routinely recommended for unexplained male infertility. (A) ART routinely offered included IVF (blue; *n*  = 55); ICSI (orange; *n*  = 28); ICSI and surgical sperm retrieval (SSR) (peach; *n*  = 11) and donor sperm (grey; *n*  = 5). Notably, some indicated individualised treatment recommendations according to semen analysis results (pale yellow; *n*  = 6) or partner/couple characteristics (green; *n*  = 6). (B) Use of empirical medical therapy (EMT) for unexplained male infertility. EMT used routinely by 48% of clinicians, including clomiphene or tamoxifen (blue; *n*  = 37), letrozole (orange; *n*  = 2), combined follicle-stimulating hormone (FSH) and human chorionic gonadotrophin (HCG) injections (grey; *n*  = 7), testosterone (yellow; *n*  = 4) and other (red; *n*  = 4). EMT not routinely used by 52% of respondents (green; *n*  = 58). (C) Vitamin and dietary supplements (VDS) for unexplained male infertility. Respondents reported recommending VDS never (blue; *n*  = 14), not routinely (orange; *n*  = 25), sometimes (grey; *n*  = 51) or always (yellow; *n*  = 22).

ART specialists were asked what fertility treatment they usually recommended for unexplained male infertility (Fig. 4A). As expected, responses spanned ART offerings, including intrauterine insemination (IUI) (0.9%), *in vitro* fertilisation (49.1%), intracytoplasmic sperm injection (ICSI) (34.8%), either alone (25.0%) or combined with surgical sperm retrieval (9.8%), or treatment using donor sperm (4.5%). Although the questionnaire asked clinicians to select an option that best represented their routine practice, there were also a variety of free text responses. These could be grouped into two themes, one where fertility treatment recommended was dependent on semen analysis abnormality and the other where treatment recommendations also considered the female partner or couple’s circumstances and previous treatment attempts. Specialists were also asked about medical treatment for unexplained male infertility (Fig. 4B). Around half (51.8%) of clinicians did not use EMT for male infertility. Those who did were most likely to prescribe clomiphene citrate (Clomid) or tamoxifen (33.0%). However, clinicians also reported the use of empirical Letrozole (1.8%) and combined follicle-stimulating hormone (FSH) and human chorionic gonadotrophin (6.3%). Notably, 3.6% of respondents reported routine use of testosterone, despite the resultant suppression of endogenous testosterone production and the risk of impaired spermatogenesis. Specialists were asked whether they recommended fertility vitamins and dietary supplements (VDS) for unexplained male infertility. Responses demonstrated clinical equipoise, with 19.6% always recommending VDS, 44.6% usually recommending VDS, 23.2% not usually recommending VDS and 12.5% never recommending VDS.

The questionnaire also asked clinicians about lifestyle and dietary changes discussed with men in preparation for fertility treatment and pregnancy (Supplementary Fig. 2). Responses demonstrated high levels of opportunistic delivery of health advice. Smoking cessation and alcohol reduction were routinely mentioned by 93% of specialists during consultations, with 73% advising regular exercise and 72% advising fruit, vegetables and a healthy diet. Modification of consumption of caffeine and advice regarding sugar reduction was mentioned less often (30% and 31%, respectively). Free text responses also included advising weight loss where appropriate, cessation of recreational drug use and avoiding exposure to extreme heat.

Lastly, we sought to explore clinic support for men and asked about the provision of fertility and/or genetic counselling. All clinics offered counselling. Uptake of fertility and/or genetic counselling was compulsory for all patients in 23.2% (26/112), required only in certain circumstances, for example, fertility preservation and treatment using donor gametes, in 25.9% (29/112) and entirely optional in 26.8% (30/112) clinics. Of note, the remaining 24.1% (27/112) of respondents reported counselling only being compulsory for women.

## Discussion

The discussion of our results is presented in the spirit of a debrief. Team debriefs are commonly used in medicine to evaluate clinical or situational performance. The structure of a basic debrief is to systematically consider what went well, what could be improved and what went badly and then subsequently focus on one or two learning points to carry forwards.

First, what do fertility specialists do well? Education regarding the potential effects of behavioural, environmental (including occupational exposures) and lifestyle factors on fertility is important to empower patients. Questionnaire responses demonstrated universally high levels of opportunistic delivery of general health and fertility-specific advice. Interaction with fertility specialists, therefore, represents an important moment for sharing medical advice and key personal health messages for men. Survey responses also demonstrated patient-centred clinical care. When clinicians were asked to indicate their usual treatment for male infertility, it was notable that a theme of personalised medicine emerged alongside the spectrum of ART treatment recommended. This included treatment recommendations that took into consideration the female partner or couple’s circumstances, including previous treatment (successful or otherwise), as well as the concept of recommending fertility treatment based on the severity of semen analysis abnormality. Clinics also universally offered fertility counselling and support, which is important because men with infertility commonly experience psychological distress that impacts their quality of life (Jacob *et al.* 2021).

Secondly, what could be improved? While hypogonadotrophic hypogonadism is a well-defined condition that responds to hormonal therapy, there is a lack of good quality evidence supporting the use of hormone treatment for unexplained male infertility. Similar to previously published studies (Buhling *et al.* 2020), antioestrogenic drugs (clomiphene citrate, tamoxifen) were the most prevalent EMTs used for unexplained male infertility, by around half of the respondents. Clomiphene citrate and tamoxifen block negative feedback at the level of the hypothalamus and pituitary, enhancing the secretion of luteinising hormone and FSH, as well as downstream testosterone synthesis and spermatogenesis. Whether this has any place in the management of male infertility with apparently normal hypothalamic–pituitary–testicular axis is less clear. The typical prescribed therapeutic dose is clomiphene citrate 25 mg daily. Higher dosages may cause hypothalamic–pituitary–gonadal downregulation. Notably, although improved semen characteristics have been reported in several studies, a limited effect on LBR has been reported to date (Chua *et al.* 2013, Willets *et al.* 2013). The use of nutraceuticals and VDS by infertile men is widespread (Martins da Silva 2019), despite currently limited scientific evidence of benefit to conception and live birth (Smits *et al.* 2019, Schisterman *et al.* 2020, Steiner *et al.* 2020). Survey responses were spread from never to always recommending VDS and demonstrated genuine clinical equipoise. In the absence of further large-scale, randomised placebo-controlled studies examining the effect of EMT and VDS on pregnancy and live birth in men with unexplained infertility, it is difficult to see how we can move forward from this position.

Thirdly, what do fertility specialists do badly? One very notable feature of the questionnaire responses is the generally poor clinical assessment of the man. Forty-one percent of clinicians (45/110) report taking only a brief medical history and 24% of clinicians reported that they never routinely examined male patients. Although honest, this is hugely disappointing and something that must be urgently addressed by our speciality, not least because clinical history and examination are key tools for diagnosis, appropriate investigation and management. Recent work has also identified associations between male infertility and various malignancies (Hanson *et al.* 2018, Behboudi-Gandevani *et al.* 2021). For example, infertile men are reported to be at least three times more likely to develop testicular cancer (Walsh *et al.* 2009, Del Giudice *et al.* 2020). Indeed, around 1% of cases presenting with male infertility have been reported to harbour a more serious or potentially fatal medical condition, including cancer, and also endocrinopathies, systemic disease and genetic syndromes (Honig *et al.* 1994). Comprehensive history taking and physical examination have the ability to detect more serious or potentially life-threatening conditions and should be approached bearing this in mind. It is also disheartening to see a small proportion of fertility specialists recommending testosterone for male infertility. Certainly, exogenous testosterone should not be used to attempt to improve sperm production as it suppresses endogenous testosterone production and therefore has a negative effect on spermatogenesis.

Lastly, what should be the focus of learning points to carry forwards? Detailed evaluation of infertile men, including clinical history and examination, would appear to be a key recommendation. The link between infertility and malignancy (primarily testicular and prostate cancer) certainly highlights the importance of immediate evaluation (Del Giudice *et al.* 2020) but perhaps also raises a bigger question about long-term follow-up for infertile men and the need to determine strategies for this. Another key learning point relates to issues encountered around male fertility assessment, specifically diagnostic semen analysis, which was reported by 54% of fertility specialists. While various reasons were cited, embarrassment, cultural and social stigma was a factor commonly reported (24%) to hinder men from producing or submitting a sample for fertility assessment. This was also a shared experience across geographical locations and would certainly account for the interest and demand for home fertility testing. The integration of microfluidics and advances in smartphone capabilities, particularly camera and optical sensing accessories, have not only made remote semen quality testing possible but also accessible to people in both developed and developing countries. Several point-of-care systems have been reported to provide a highly accurate evaluation of semen based on the World Health Organization guidelines (Kanakasabapathy *et al.* 2017), indicating the potential for technological solutions that may be acceptable to both patients and clinics. In the meantime, our speciality must be more cognisant of the lived experience of male infertility, appreciate the personal shame and embarrassment experienced by some men and continue to take opportunities as fertility specialists to deliver astute clinical care and to destigmatise male infertility. Infertility is not solely a female problem, and we should not just be women’s health specialists.

## Supplementary Material

Supplementary table 1 SurveyMonkey® questionnaire used in this study

Supplementary figure S1 Number of ART treatment cycles performed by respondent’s clinics per year (less than 100 (blue) n=39; 100 – 250 (orange) n=11; 250 – 500 (grey) n=14; over 500 (yellow) n=48).

Supplementary figure S2 Medical advice regarding diet and life exposures and unexplained male infertility. Others (n=6) included weight reduction (if appropriate); recreational drug use including anabolic steroids; exposure to extreme heat, including saunas, hot tubs and choice of underwear.

## Declaration of interest

S M D S is Associate Editor of Reproduction and Fertility. S M D S was not involved in the review or editorial process for this paper, on which she is listed as an author. The other authors declare no conflict of interest.

## Funding

This study did not receive any specific grant from any funding agency in the public, commercial or not-for-profit sector.

## Data availability statement

The data underlying this article will be shared on reasonable request to the corresponding author.

## Author contribution statement

S M D S was responsible for study concept. N P O, L C E and S M D S designed the questionnaire and acquired data. N P O and S M D S analysed and interpreted the data. N P O and S M D S drafted the manuscript. All authors revised and contributed to final manuscript, approved the version submitted for publication and agree to be accountable for all aspects of the work presented.
