# Supplementary material for: Male infertility: what on earth is going on? Pilot international questionnaire study regarding clinical evaluation and fertility treatment for men
Source: Reprod Fertil. 2022 Sep 26;3(3):207–15. doi: 10.1530/RAF-22-0033 (PMC9578063; doi:10.1530/RAF-22-0033)
Supplement: Supplementary table 1 SurveyMonkey® questionnaire used in this study [file supplementary_table_1.pdf]

Supplementary Table 1

1. What country do you work in?

2. How many ART treatment cycles does your clinic perform per year?			
<100	100 - 250	250 - 500	>500

3. How are male patients routinely evaluated?
Take a brief medical history only
Take a detailed medical history from a fertility perspective, including life exposures
Take a brief history and sometimes examine him
Take a detailed medical history from a fertility perspective and sometimes examine him
Take a brief history and usually examine him
Take a detailed medical history from a fertility perspective and usually examine him
Other (please specify)

4. Do you experience any issues getting men to undertake fertility testing (diagnostic semen analysis)?
No – there are no issues
Yes – because men are sexually active and assume they have no fertility problem
Yes – because men have previously fathered a pregnancy and assume they have no fertility problem
Yes – because men are not comfortable with producing / submitting a sample for analysis
Yes – because men assume that infertility is a woman’s issue
Other (please specify)

5. Do you recommend fertility vitamin and dietary supplements for unexplained male infertility?
Yes - always
Yes - sometimes
Not routinely
Yes - only when clinically needed
Never

6. What medical treatment do you routinely recommend for unexplained male infertility?
Clomiphene (clomid) or tamoxifen
Letrozole
FSH and HCG combined
HCG
Testosterone
None of the above
Other (please specify)

7. Do you advise men to make lifestyle and dietary changes in preparation for fertility treatment and pregnancy? (please tick all that apply)

Alcohol reduction
Smoking cessation
Caffeine reduction
Sugar reduction
Advice about regular exercise
Advice about fruit, vegetables and healthy diet
None of the above
Other (please specify)

8. What fertility treatment do you usually recommend for unexplained male infertility?
IVF
ICSI
ICSI and surgical sperm retrieval (SSR)
Donor sperm
Other (please specify)

9. Is fertility and/or genetic counselling compulsory for patients?
Yes – but only women
Yes – but only men
Yes – for both
Yes – but only in certain circumstances, for example fertility preservation, donor gamete treatment
No - it is optional

10. Do you think it is acceptable for a clinic to charge patients for donor sperm that has been altruistically donated? (you may tick more than one option)
Yes - so patients appreciate that it is valuable
Yes – because it makes a profit for the clinic
Yes – because it generates funds for the running of the clinic
Yes – but only to cover costs
No – clinics shouldn’t make a profit on something that was altruistically donated
No - other reason
Other (please specify)
