# Supplementary figures and images for: Male infertility: what on earth is going on? Pilot international questionnaire study regarding clinical evaluation and fertility treatment for men
Source: Reprod Fertil. 2022 Sep 26;3(3):207–15. doi: 10.1530/RAF-22-0033 (PMC9578063; doi:10.1530/RAF-22-0033)

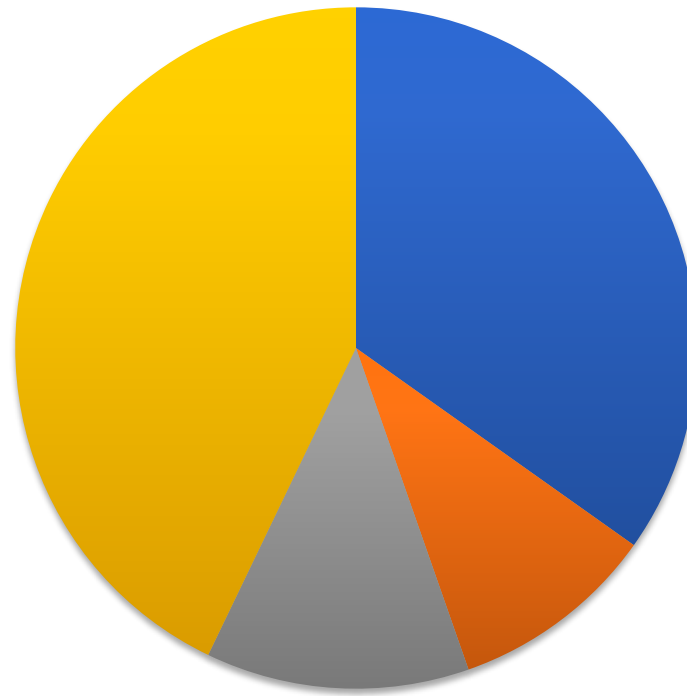

■ LESS THAN 100 ■ 100 TO 250 ■ 250 TO 500 ■ ABOVE 500

Supplement: Supplementary figure S1 Number of ART treatment cycles performed by respondent’s clinics per year (less than 100 (blue) n=39; 100 – 250 (orange) n=11; 250 – 500 (grey) n=14; over 500 (yellow) n=48). [file supplementary_figure_1.pdf]

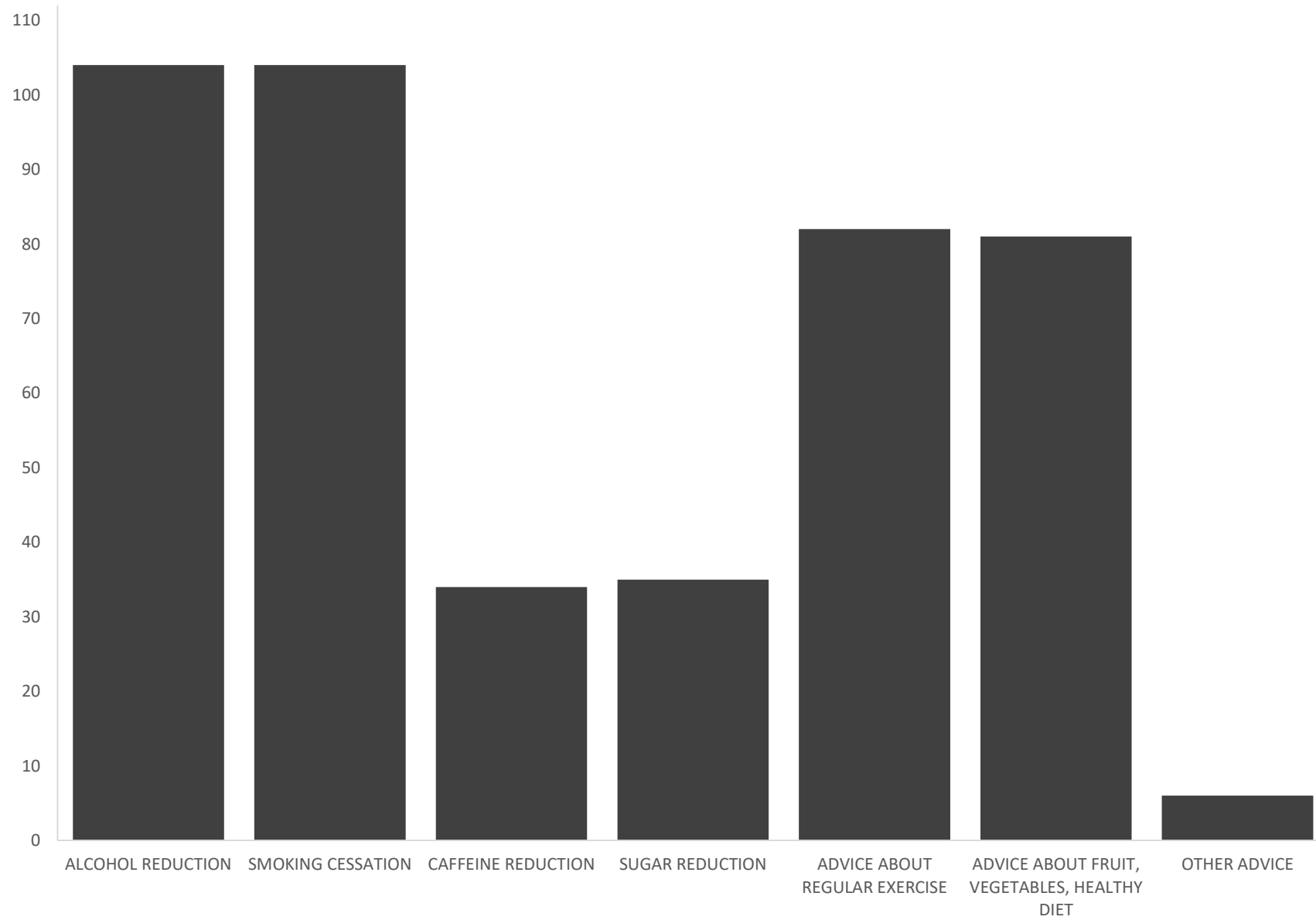

Supplement: Supplementary figure S2 Medical advice regarding diet and life exposures and unexplained male infertility. Others (n=6) included weight reduction (if appropriate); recreational drug use including anabolic steroids; exposure to extreme heat, including saunas, hot tubs and choice of underwear. [file supplementary_figure_2.pdf]
